# Control of CD4^+^ T cells to restrain inflammatory diseases via eukaryotic elongation factor 2 kinase

**DOI:** 10.1038/s41392-023-01648-5

**Published:** 2023-10-24

**Authors:** Hao-Yun Peng, Liqing Wang, Jugal Kishore Das, Anil Kumar, Darby J. Ballard, Yijie Ren, Xiaofang Xiong, Paul de Figueiredo, Jin-Ming Yang, Jianxun Song

**Affiliations:** 1 Department of Microbial Pathogenesis and Immunology, Texas A&M University Health Science Center, Bryan, TX 77807 USA; 2 Department of Biochemistry and Biophysics, Texas A&M University, College Station, TX 77843 USA; 3 Department of Veterinary Pathobiology, Texas A&M University, College Station, TX 77845 USA; 4 Department of Toxicology and Cancer Biology, Department of Pharmacology and Nutritional Science, and Markey Cancer Center, University of Kentucky College of Medicine, Lexington, KY 40536 USA

**Keywords:** Adaptive immunity, Experimental models of disease

## Abstract

CD4^+^ T cells, particularly IL-17-secreting helper CD4^+^ T cells, play a central role in the inflammatory processes underlying autoimmune disorders. Eukaryotic Elongation Factor 2 Kinase (eEF2K) is pivotal in CD8^+^ T cells and has important implications in vascular dysfunction and inflammation-related diseases such as hypertension. However, its specific immunological role in CD4^+^ T cell activities and related inflammatory diseases remains elusive. Our investigation has uncovered that the deficiency of eEF2K disrupts the survival and proliferation of CD4^+^ T cells, impairs their ability to secrete cytokines. Notably, this dysregulation leads to heightened production of pro-inflammatory cytokine IL-17, fosters a pro-inflammatory microenvironment in the absence of eEF2K in CD4^+^ T cells. Furthermore, the absence of eEF2K in CD4^+^ T cells is linked to increased metabolic activity and mitochondrial bioenergetics. We have shown that eEF2K regulates mitochondrial function and CD4^+^ T cell activity through the upregulation of the transcription factor, signal transducer and activator of transcription 3 (STAT3). Crucially, the deficiency of eEF2K exacerbates the severity of inflammation-related diseases, including rheumatoid arthritis, multiple sclerosis, and ulcerative colitis. Strikingly, the use of C188-9, a small molecule targeting STAT3, mitigates colitis in a murine immunodeficiency model receiving eEF2K knockout (KO) CD4^+^ T cells. These findings emphasize the pivotal role of eEF2K in controlling the function and metabolism of CD4^+^ T cells and its indispensable involvement in inflammation-related diseases. Manipulating eEF2K represents a promising avenue for novel therapeutic approaches in the treatment of inflammation-related disorders.

## Introduction

Inflammation-related disorders, including rheumatoid arthritis, ulcerative colitis, and multiple sclerosis, have experienced a significant increase in incidence over recent decades, particularly in Western countries. Rheumatoid arthritis afflicts approximately 1% of adults, totaling 1.3–1.5 million individuals in the United States each year.^1,2^ Ulcerative colitis in North America is witnessing an annual increase of 0.0192% in new cases,^3^ while multiple sclerosis affects roughly 2.3 million individuals globally.^4^ Patients with inflammatory diseases often experience a decreased quality of life. For example, rheumatoid arthritis causes musculoskeletal pain and physical disability,^1,2^ ulcerative colitis presents with systemic symptoms such as weight loss and bloody diarrhea,^5,6^ and individuals with multiple sclerosis commonly report numbness and difficulty walking.^4^ The primary driver of the pathogenesis of these debilitating diseases is aberrant inflammatory reactions, characterized by inflammation occurring in distinct regions of the body. This includes joint inflammation in rheumatoid arthritis, inflammation of the digestive tract in ulcerative colitis, and central nervous system inflammation in multiple sclerosis. This dysregulated inflammation is characterized by an imbalance of immune cells and increased production of pro-inflammatory cytokines. CD4^+^ T cells, particularly their subsets, the IL-17-secreting helper CD4^+^ T cells (Th17 cells), are a major source of pro-inflammatory cytokines, such as IL-6, IL-17A, and IL-17F, and are believed to play an essential role in inflammatory disorders.^7–9^ CD4^+^ T cells play a pivotal role in immune responses, while the pro-inflammatory cytokines they promote drive inflammation and facilitate the recruitment of other immune cells. Emerging research indicates that elevated populations of Th17 cells play a pivotal role in driving inflammation in individuals with arthritis,^10,11^ those afflicted by multiple sclerosis,^12,13^ and in experimental models simulating diseases associated with ulcerative colitis.^14–16^ In rheumatoid arthritis, Th17 cells are facilitated in the inflamed joints by the expression of CCL20, which serves as the ligand for a chemokine receptor expressed by Th17 cells.^11^ Th17 cells can penetrate the blood-brain barrier and disrupt the myelin in the central nervous system of individuals with multiple sclerosis.^12,13^ Autoreactive Th17 cells shape the immune microenvironment within the intestinal mucosa through their pro-inflammatory cytokine production.^16^ Enhanced comprehension of T cell functionality and its regulatory mechanisms in the context of inflammation holds the promise of facilitating the development of innovative and efficacious strategies for addressing hyperinflammatory diseases.

Eukaryotic elongation factor 2 kinase (eEF2K) is a protein kinase that serves a fundamental role in various cellular processes. Its activation depends on calcium ions and calmodulin.^17^ This kinase exerts control over protein synthesis by phosphorylating eEF-2 on Thr-56, negatively regulating the elongation step.^17^ Increasingly compelling data indicates that eEF2K might control diverse cellular processes and metabolism across different cell types, including immune cells.^17^ eEF2K promotes the proliferation and viability of macrophages, especially within atherosclerotic plaques, where its function becomes heightened in response to oxidized low-density lipoprotein and increased calcium ion concentrations.^18^ Additionally, it has been demonstrated that CD8^+^ T cells lacking eEF2K exhibit elevated metabolic profiles and are crucial for maintaining the functionality of cytotoxic CD8^+^ T cells.^19^ Defining the role of eEF2K in various cellular processes presents a promising objective for addressing multiple pathological conditions.

eEF2K has also emerged as a key regulator in inflammation-related diseases, including hypertension, vascular inflammatory responses,^20,21^ and neurodegenerative diseases.^17^ Increased eEF2K expression has been detected in the aorta of spontaneously hypertensive rats, a well-established model for hypertension characterized by the natural development of elevated blood pressure with age.^22^ Evidence also suggests that eEF2K modulates oxidative-stress-dependent vascular inflammation, further contributing to the onset of hypertension.^20^ Moreover, an experimental study by Kameshima et al. has proposed that eEF2K promotes vascular structural remodeling through mediating the reactive oxygen species (ROS) pathway, partially influencing the development of pulmonary arterial hypertension.^23^ Nevertheless, the functions of eEF2K in other inflammatory conditions like rheumatoid arthritis, multiple sclerosis, and ulcerative colitis, remain unclear. Consequently, new insights into the biology of eEF2K in inflammatory diseases are needed. While we have recently reported on the role of eEF2K in regulating the fate and antitumor immunity of CD8^+^ T cells,^19^ the full impact of eEF2K on effector T cells, especially CD4^+^ effector T cells, remains to be fully defined. Given the existing knowledge limitations in these areas, the present study was devised to explore the connection between eEF2K, CD4^+^ T cells, and inflammatory diseases.

In this research, we explored the importance of eEF2K in influencing the activities of CD4^+^ T cells, encompassing their functionality and metabolic processes. Our results have uncovered that the absence of eEF2K in effector CD4^+^ T cells inclines them towards adopting a Th17 phenotype. We have revealed that eEF2K’s functional role in the control of CD4^+^ T cells is mediated through the modulation of mitochondrial bioenergetics and the activity of the Th17 master transcription factor, STAT3. Furthermore, we have documented that the absence of eEF2K worsens the occurrence of ulcerative colitis, multiple sclerosis, and the onset of rheumatoid arthritis in murine models, primarily by amplifying the production of pro-inflammatory cytokines. This study carries significant implications in the prevention of inflammatory diseases and offers novel insights into the treatment of autoimmune disorders.

## Results

### Activated CD4^+^ T cells lacking eEF2K lead to a Th17 profile and display dysfunctional phenotypes

**Fig. 1:**
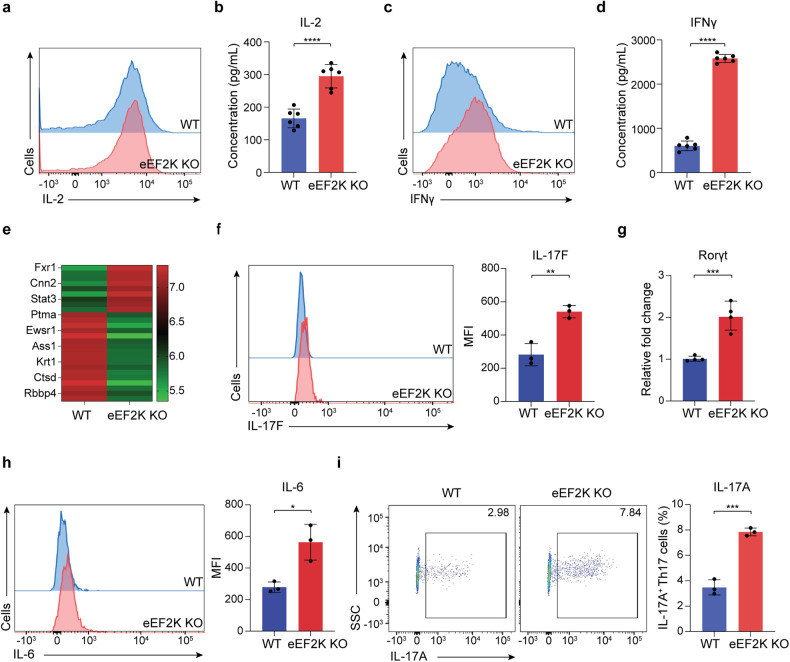
eEF2K-deficient activated CD4^+^ T cells lead to a Th17 profile and dysfunction phenotypes. CD4^+^ T cells were isolated from both wild-type (WT) and eEF2K-knockout (KO) C57BL/6 mice after three days of in vitro stimulation on plates coated with anti-CD3 monoclonal antibody (mAb), in combination with soluble anti-CD28 mAb. **a** Representative result of the intracellular staining analysis of IL-2 in the WT and eEF2K KO effector CD4^+^ T cells. **b** Result of ELISA for IL-2 in the supernatants of the CD4^+^ T cells, stimulated for 48 h, was shown in technical replicates with the standard deviation (*n* = 6). **c** Representative result of the intracellular staining analysis of IFNγ in the WT and eEF2K KO effector CD4^+^ T cells. **d** Result of ELISA for IFNγ in the supernatants of the CD4^+^ T cells, with stimulation for 48 h, were shown in technical replicates with the standard deviation (*n* = 6). **e** LC/MS-MS proteomics spectral analysis of inflammation-related proteins. The result of post-activated WT and eEF2K KO CD4^+^ T cells was in the format of heatmap. **f** Intracellular FACS analysis of IL-17F in the WT and eEF2K KO effector CD4^+^ T cells on day 3. Summarized MFI (*n* = 3) were shown in the bar graph. **g** mRNA level of RORγt from WT and eEF2K KO CD4^+^ T cells were examined through qPCR (*n* = 4: technical replicates). **h** Intracellular FACS analysis of IL-6 in the WT and eEF2K KO effector CD4^+^ T cells on day 3. The results of FACS analysis, summarized MFI (*n* = 3), in the histogram format were shown in the bar graph, with the standard deviations. **i** Effector CD4^+^ T cells were stimulated with Th17-associated cytokines for 4 days—intracellular FACS analysis of IL-17A in the WT and eEF2K KO Th17 cells. The percentages of cells were indicated in each quadrant, and the results of FACS analysis in the flow chart were shown in the bar graph, with the standard deviations. Summary data were presented as mean ± SD, derived from three independent experiments. *P < 0.05; **P < 0.01; ***P < 0.005; ****P < 0.001, unpaired *t*-test

In studying the phenotypic effects of eEF2K on CD4^+^ T cells, we initially validated that CD4^+^ T cells isolated from the spleen of eEF2K-knockout (eEF2K KO) mice were in fact deficient of eEF2K (Supplementary Fig. 1a). Effector eEF2K KO CD4^+^ T cells were hyperactivated as indicated by their significantly higher expression of CD69, compared to WT CD4^+^ T cells; however, no distinction was observed between naive WT and eEF2K knockout CD4^+^ T cells (Supplementary Fig. 1b). To assess the effects of eEF2K deficiency on the proliferative capacity of CD4^+^ T cells, we measured the percentage of dividing cells after incubation with Carboxyfluorescein Diacetate Succinimidyl Ester (CFSE). While there was an initial surge in cell division among eEF2K knockout CD4^+^ T cells during days 1–3 (Supplementary Fig. 1c), their growth rate decreased beyond day 4 (Supplementary Fig. 1d). To evaluate the impact of eEF2K deficiency on the function of CD4^+^ T cells, we quantified the secretion of IL-2 and IFNγ, which are the primary cytokines produced by CD4^+^ T cells. The number of IL-2- and IFNγ-producing CD4^+^ T cells markedly increased in eEF2K KO CD4^+^ T cell cultures, as analyzed with flow cytometry and ELISA (Fig. 1a–d). We conducted a global proteomics analysis on eEF2K KO and WT CD4^+^ T cells using liquid chromatography-tandem mass spectrometry (LC-MS/MS). The LC/MS-MS proteomics spectral analysis unveiled changes in the expression profiles of inflammation-related proteins in the absence of eEF2K (Fig. 1e). Additionally, increased secretion of pro-inflammatory cytokines, including IL-6 and IL-17F, were observed in eEF2K KO CD4^+^ T cells (Fig. 1f, h). The expression of Rorγt, one of the Th17 key transcription factors, was found to be higher in eEF2K KO CD4^+^ T cells (Fig. 1g). We examined the populations of all CD4^+^ T cell subsets, including Th1, Th2, Treg, and Th17 cells. In eEF2K KO cell cultures, there was an increase in the populations of Th1 and Th17 cells, while the populations of Th2 and Treg cells decreased (Supplementary Fig. 1e–h). We verified a significant increase in the count of IL-17A-producing cells within the eEF2K KO Th17 cell cultures following activation with a range of cytokines, including IL-4, IL-6, IL-23, and TGF-β (Fig. 1i). Surprisingly, the cytokine secretions from Treg cells exhibited no statistically significant alterations (Supplementary Fig. 1i, j). These findings suggest that the absence of eEF2K has the potential to give rise to an inflammatory microenvironment. Together, Fig. 1 and Supplementary Fig. 1 suggest an important role of eEF2K in maintaining CD4^+^ T cell activities, including survival and proliferative capacity. The absence of eEF2K may potentially lead to an inflammatory environment determined by dysfunctional CD4^+^ T cells and abnormal cytokine secretions.

### The absence of eEF2K in CD4^+^ T cells leads to changes in their metabolism and mitochondrial function

**Fig. 2:**
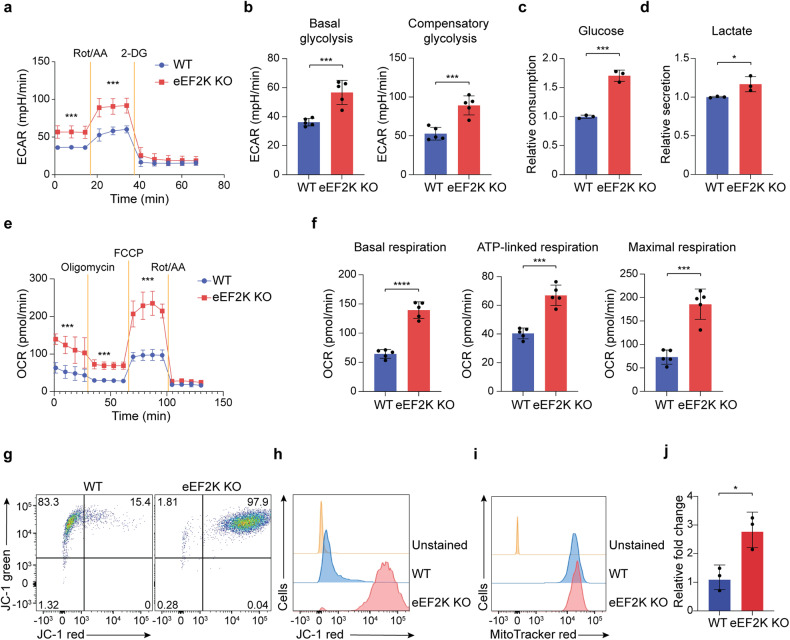
Loss of eEF2K in CD4^+^ T cells alters their metabolism and mitochondrial function. Naive CD4^+^ T cells from both WT and eEF2K KO mice were subjected to a three-day stimulation period using anti-CD3 mAb and anti-CD28 mAb. **a** Cells were treated with a Rotenone/Antimycin A mix to block mitochondrial activities and 2-DG to inhibit glycolysis. The extracellular acidification rate (ECAR) from the Seahorse glycolytic rate assay determined the glycolytic rate. **b** Basal glycolysis and the compensatory glycolysis of WT and eEF2K KO CD4^+^ T cells in **a** were shown (*n* = 5: technical replicates). **c** Quantification of relative glucose consumption in WT and eEF2K KO CD4^+^ T cells on day 3, using Glucose Uptake-Glo assay. (*n* = 3: technical replicates). **d** Quantification of relative lactate consumption in WT and eEF2K KO CD4^+^ T cells on day 3, examined with Lactate-Glo assay. (*n* = 3: technical replicates). **e** Oxygen consumption rate (OCR) from the Seahorse Mito Stress test was determined for mitochondrial stress and oxidative phosphorylation in WT and eEF2K KO effector CD4^+^ T cells on day 3 (*n* = 5: technical replicates). **f** WT and eEF2K KO CD4^+^ T cells are treated with Oligomycin, Carbonyl cyanide-4 (trifluoromethoxy) phenylhydrazone (FCCP), and Rotenone/Antimycin A mix in order to block ATP synthase, to collapse proton gradient, and to inhibit mitochondria activities. Basal respiration, ATP-linked respiration, and maximal respiration were determined by the Seahorse Mito-Stress test of WT and eEF2K KO CD4^+^ T cells (**e**), and the data were shown with the standard deviations (*n* = 5: technical replicates). FACS results of mitochondrial membrane potential (MMP) in WT and eEF2K KO CD4^+^ T cells were shown on day 3, measured with JC-1 dye (**g**). JC-1 red indicates red fluorescent J-aggregates and potential mitochondrial hyperpolarization. The percentages of cells were indicated in each quadrant, and JC-1 red was shown in the histogram format (**h**). Mitochondrial mass of WT and eEF2K KO CD4^+^ T cells were examined by FACS staining with mitoTracker Red (**i**) and by qRT-PCR (**j**) (*n* = 3; technical replicates). Summary data were presented as mean ± SD, derived from three independent experiments. *P < 0.05; **P < 0.01; ***P < 0.005; ****P < 0.001, unpaired *t*-test

Upon T cell receptor (TCR) engagement, T cells undergo both translational and transcriptional alterations, enabling vigorous metabolic processes that facilitate the secretion of cytokines.^24,25^ Furthermore, inflammation is driven by cellular metabolism, encompassing glycolysis and oxidative phosphorylation (OXPHOS).^26,27^ Given eEF2K’s crucial role in controlling cellular metabolism,^28^ we subsequently assessed the impact of this kinase on energy metabolism in CD4^+^ T cells. We treated WT or eEF2K KO CD4^+^ T cells with rotenone, antimycin A, and 2-DG, then determined the glycolytic activity in these cells. Both the basal and compensatory glycolytic activities, as evidenced by the extracellular acidification rate (ECAR), were notably elevated in eEF2K KO CD4^+^ T cells compared to their WT counterparts. (Fig. 2a, b). Consistent findings were observed in glucose uptake and lactate assays, revealing elevated glucose uptake (Fig. 2c) and increased lactate production (Fig. 2d) in eEF2K KO CD4^+^ T cells compared to the control group. Furthermore, CD4^+^ T cells from eEF2K KO mice exhibited increased oxidative phosphorylation (OXPHOS) characteristics, including the oxygen consumption rate (OCR), basal respiration, ATP-linked respiration, and maximal respiration, compared to those from WT mice following treatment with oligomycin, FCCP, rotenone, or antimycin A (Fig. 2e, f). Subsequently, we measured indicators of mitochondrial function that promote the production of pro-inflammatory cytokines. The mitochondrial membrane potential (MMP), evaluated using JC-1 dye staining, was found to be higher in eEF2K KO CD4^+^ T cells compared to the control group (Fig. 2g, h). Furthermore, both flow cytometry and qRT-PCR analysis revealed that the mitochondrial mass was greater in eEF2K KO CD4^+^ T cells compared to WT CD4^+^ T cells (Fig. 2i, j). Additionally, similar outcomes regarding mitochondrial activities, such as MMP and mitochondrial mass, were observed in cells cultured under Th17 cell conditions (Supplementary Fig. 2a, b). These findings indicate that CD4^+^ T cells deficient in eEF2K exhibit modified mitochondrial metabolism and function.

### The heightened production of reactive oxygen species (ROS) is positively correlated with the increased Th17 cytokine production in eEF2K-deficient CD4^+^ T cells

**Fig. 3:**
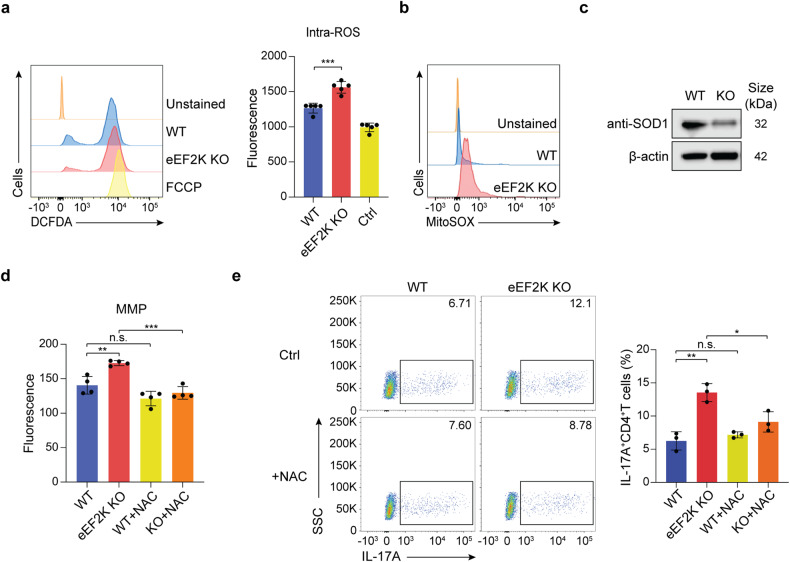
Increased ROS production is positively associated with enhanced Th17 cytokine production in eEF2K-deficient CD4^+^ T cells. Naive WT and eEF2K KO CD4^+^ T cells were stimulated with anti-CD3 mAb and anti-CD28 mAb for 3 days. **a** Cells were stained with DCFDA. Intracellular ROS production was measured by (left) flow cytometry and (right) fluorescence reader (*n* = 5; technical replicates). **b** Mitochondrial ROS production was determined in WT and eEF2K KO effector CD4^+^ T cells on day 3 by FACS staining with MitoSOX. Unstained sample as a negative control. **c** Western blot analysis of antioxidant SOD expression. **d** The average fluorescence of JC-1 red dye, indicating mitochondrial membrane potential (MMP) in WT and eEF2K KO CD4^+^ T cells on day 3, with or without the treatment of ROS inhibitor, NAC, were examined in the plate reader (*n* = 4: technical replicates). The fluorescence was detected in the orange-colored emission (590 ± 17.5 nm). Summary data were presented in the bar graph as mean ± SD. **e** Intracellular FACS analysis of IL-17A, with or without NAC, in WT and eEF2K KO CD4^+^ T cells. The percentages of cells were indicated in each quadrant, and the results of FACS analysis in the flow chart were shown in the bar graph. Summary data were presented as mean ± SD, derived from three independent experiments. *P < 0.05; **P < 0.01; ***P < 0.005; unpaired *t*-test

T cells undergo metabolic reprogramming and engage in a variety of biological processes to fulfill their energetic and biosynthetic requirements throughout their lifecycle. Since modifications in mitochondrial metabolism can lead to shifts in ROS production, this can have profound effects on CD4^+^ T cell functions and the initiation of pro-inflammatory signaling pathways,^29,30^ we next evaluated the influence of eEF2K on ROS production in CD4^+^ T cells. We conducted measurements of intracellular ROS production through a dichlorofluorescein diacetate (DCFDA)/dichlorodihydrofluorescein diacetate (H2DCFDA) assay, and we assessed mitochondrial ROS secretion using mitoSOX staining. Elevated intracellular ROS production (Fig. 3a) and mitochondrial ROS production (Fig. 3b) were observed in eEF2K KO CD4^+^ T cells on the third day of T cell activation, a pattern similar to that seen in Th17 cell culture (Supplementary Fig. 2c, d). The expression of SOD1, an antioxidant enzyme, was lower in eEF2K KO CD4^+^ T cells in comparison to the control group (Fig. 3c). Dysfunction in mitochondria has been demonstrated to lead to an elevated production of pro-inflammatory cytokines.^31^ To examine the impact of ROS production on T cell function, we employed pharmacological inhibition of ROS production in eEF2K KO CD4^+^ T cells using N-acetyl-L-cysteine (NAC), an inhibitor of ROS-dependent apoptosis.^32^ Our investigation revealed that the treatment of eEF2K KO CD4^+^ T cells with NAC significantly reduced their mitochondrial membrane potential (Fig. 3d). Notably, eEF2K KO CD4^+^ T cells but not WT CD4^+^ T cells showed decreased secretion of the Th17-associated cytokines, IL-17A and IL-17F, on day 3 following treatment with NAC (Fig. 3e and Supplementary Fig. 2e). These findings suggest that the impact of eEF2K on CD4^+^ T cell function is mediated through ROS.

### Up-regulation of STAT3 in eEF2K deficient CD4^+^ T cells is responsible for the alteration of mitochondrial function and Th17 cytokine secretions

**Fig. 4:**
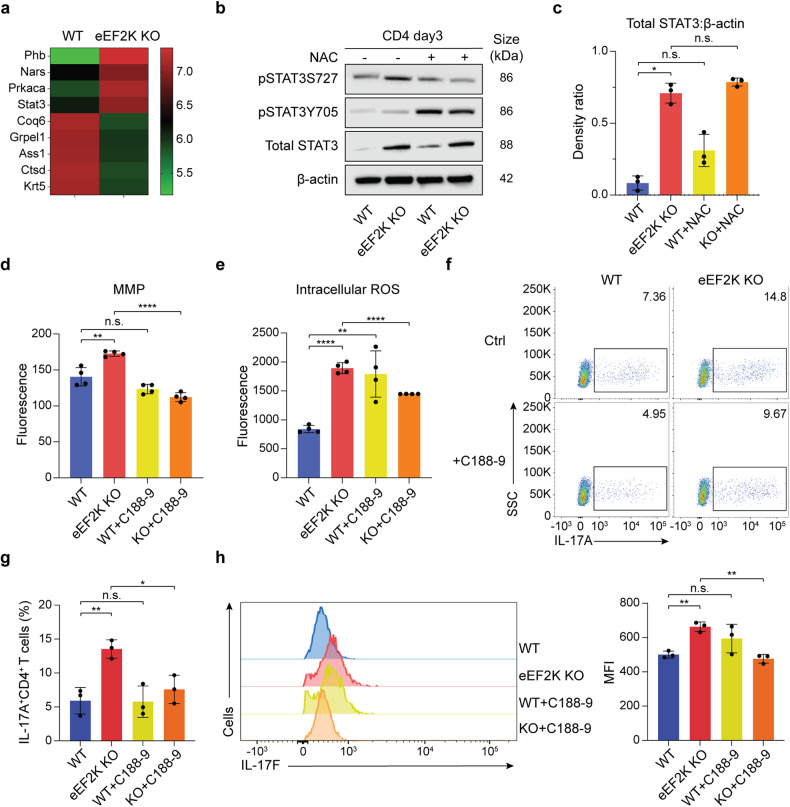
Up-regulation of STAT3 in eEF2K deficient CD4^+^ T cells is responsible for altering mitochondrial function and Th17 cytokine secretions. **a** Heat map of the associated mitochondrial proteins from WT and eEF2K KO CD4^+^ T cells. WT or eEF2K KO naive CD4^+^ T cells were stimulated with anti-CD3 mAb plus anti-CD28 mAb for 3 days and subjected to proteomics analysis. **b** Activated WT or eEF2K KO CD4^+^ T cells were grown for 3 days, treated with or without NAC, and subjected to immunoblotting. Results of the immunoblot analysis of the two phosphorylated sites, phospho (p)-STAT3 S727 and p-STAT Y705, and total STAT3 expression of WT and eEF2K KO CD4^+^ T cells. β-actin acted as the loading control. **c** Density ratio of total STAT3 /β-actin, analyzed from the immunoblot analysis of WT and eEF2K KO CD4^+^ T cells (**b**), with or without the treatment of NAC, was assayed. Summary data were presented as mean ± SD, derived from 3 independent experiments, and analyzed with paired *t*-test. **d** Result of the mitochondrial membrane potential of WT and eEF2K KO CD4^+^ T cells, with or without the C188-9 treatment. The cells were measured with JC-1 dye, and the summary data were presented as mean ± SD (*n* = 4; technical replicates). **e** Naive WT and eEF2K KO CD4^+^ T cells were stimulated with anti-CD3 mAb and anti-CD28 mAb for 3 days and then treated with the STAT3 inhibitor, C188-9, on day 3. Intracellular ROS was stained with DCFDA and analyzed using a fluorescence reader (*n* = 4: technical replicates). **f**, **g** IL-17A cytokine secretion of WT and eEF2K KO CD4^+^ T cells, with or without C188-9 treatment, was performed via intracellular staining and analyzed by flow cytometry. The percentages of cells were indicated in each quadrant, and the results of FACS analysis were shown in a bar graph (**g**) with the standard deviations (*n* = 3). **h** CD4^+^ T cells were grown and were stimulated with anti-CD3 and anti-CD28 antibodies for 3 days. Prior to intracellular staining, cells were treated with C188-9 for 1 h at 37 °C. Intracellular staining analysis of IL-17F was performed, and the summarized MFI (*n* = 3) was presented in the bar graph. Summary data were presented as mean ± SD, derived from 3 independent experiments. *P < 0.05; **P < 0.01; ***P < 0.005; ****P < 0.001, unpaired *t*-test or paired *t*-test

To explore the molecular pathway(s) linking mitochondrial alterations and CD4^+^ T cell function, we performed an LC-MS/MS proteomics analysis. This analysis found that STAT3, one of the primary transcription factors of Th17s and a regulator of the mitochondrial respiratory chain, was upregulated in eEF2K KO CD4^+^ T cells compared to the corresponding controls (Fig. 4a). Further, Western blots of the phosphorylated (p)-STAT3 and total STAT3 showed an increased level of STAT3 in eEF2K KO CD4^+^ T cells, compared to the WT CD4^+^ T cells (Fig. 4b, c). However, two essential phosphorylated sites, phosphorylation at tyrosine 705 (pY705-STAT3) and at serine 727 (pS727-STAT3), were not drastically changed (Fig. 4b). To verify whether STAT3 impacted the Th17 profile, we grew Th17 cells in vitro and performed western blot analysis using antibodies targeting STAT3 or pSTAT3 (Supplementary Fig. 3a, b). Similarly, total STAT3 but not phosphorylated sites of STAT3 level was significantly higher in eEF2K KO CD4^+^ T cells under Th17 conditions, which confirms that eEF2K mediates the Th17 profile through STAT3.

To establish the involvement of STAT3 in eEF2K-mediated regulation of mitochondrial activity and cellular function in CD4^+^ T cells, we administered a STAT3 inhibitor, C188-9, to both WT and eEF2K KO CD4^+^ T cells, and subsequently assessed MMP, ROS levels, and Th17 cytokine production. We detected a decrease in MMP in eEF2K KO CD4^+^ T cells treated with C188-9 (Fig. 4d). In eEF2K KO CD4^+^ T cells treated with C188-9, we observed reductions in both intracellular and mitochondrial ROS production (Fig. 4e and Supplementary Fig. 3c). The eEF2K knockout group exhibited higher levels of IL-17A and IL-17F cytokine production compared to the WT CD4^+^ T cell group (Fig. 4f–h); Treatment with C188-9 markedly reduced the production of IL-17A and IL-17F in eEF2K KO CD4^+^ T cells, whereas it had no impact on the production of these cytokines in WT CD4^+^ T cells (Fig. 4f–h). These findings indicate that STAT3 serves as a pivotal mediator of eEF2K-regulated mitochondrial functions and the functionality of CD4^+^ T cells.

To verify that STAT3 acted upstream of ROS, we treated WT and eEF2K KO CD4^+^ T cells with NAC. eEF2K KO CD4^+^ T cells under Th17 conditions showed similarly to CD4^+^ T cells in normal conditions, as there was no difference with or without NAC treatment (Fig. 4b, c and Supplementary Fig. 3a, b). Together, these data supported that eEF2K deficient CD4^+^ T cells upregulate STAT3, resulting in mitochondrial dysfunction and redox imbalance, further leading to pro-inflammatory Th17 cytokine secretions.

### Loss of eEF2K exacerbates inflammation-related diseases

**Fig. 5:**
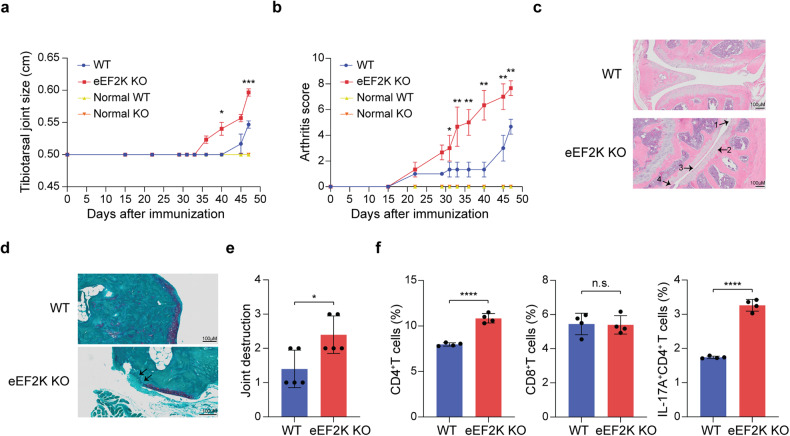
The absence of eEF2K exacerbates rheumatoid arthritis in the collagen-induced arthritis (CIA) model. On day 0 and day 15, both WT and eEF2K KO mice received injections of an emulsion containing complete Freund’s adjuvant (CFA) and bovine type II collagen. Tibiotarsal joint size and arthritis scores were assessed every 2-3 days by two independent investigators, who conducted the evaluations without knowledge of the experimental groups. Once the arthritis score criteria were met, the mice afflicted with CIA were euthanized for further analysis. **a** Tibiotarsal joint size of WT and eEF2K KO mice with/without CIA were measured (*n* = 5 mice in each group). **b** The quantification of arthritis score (*n* = 5) mice in each group. **c** H&E staining of knee joint isolated from WT and eEF2K KO mice with CIA. Arrows showed (1) cartilage damage, (2) chondrocyte hypertrophy, (3) infiltration of inflammatory cells, and (4) synovial lining hyperplasia in the eEF2K KO mice with CIA. Scale bar, 100 μm. **d** Representative images of Safranin-O-stained knee joint sections from WT and eEF2K KO mice with CIA. The purple area represents the cartilage. Arrow pointed at the loss of cartilage. Scale bar, 100 μm. **e** Joint destruction from Safranin-O-stained knee joint sections was quantified based on **d** in each group. **f** Representative results of the surface and intracellular FACS analysis of CD4, CD8, and IL-17A in WT and eEF2K KO T cells from the spleens of the mice with CIA. Summary data were presented as mean ± SD, derived from 3 independent experiments. *P < 0.05; **P < 0.01; ***P < 0.005; ****P < 0.001, unpaired *t*-test

The deficiency of eEF2K promotes a pro-inflammatory microenvironment, which is closely linked to autoimmune disorders like rheumatoid arthritis—a condition characterized by CD4^+^ T cell hyperactivation,^33,34^ we next delved into whether the absence of eEF2K in CD4^+^ T cells has any influence on the development of rheumatoid arthritis. We employed a collagen-induced arthritis (CIA) mouse model, where the disease was induced by injecting Complete Freund’s adjuvant and type II collagen into both WT and eEF2K KO mice (Supplementary Fig. 4a). We monitored the size of the tibiotarsal joints and conducted arthritis scoring at intervals of 2-3 days for a duration of 35 days following the onset of arthritis. The tibiotarsal joint sizes of the eEF2K KO mice with CIA were significantly larger than WT mice with the CIA (Fig. 5a and Supplementary Fig. 4b). The eEF2K KO mice with CIA also showed a higher arthritis score (Fig. 5b). Furthermore, histological analysis of the knee joint by H&E staining showed (1) cartilage damage (2) chondrocyte hypertrophy (3) infiltration of inflammatory cells and (4) synovial lining hyperplasia in the eEF2K KO group than in controls (Fig. 5c). Safranin O staining showed severe inflammation characterized by heavier loss of cartilage in the eEF2K KO mice with CIA (Fig. 5d, e). We did not find any difference between WT and eEF2K KO CD8^+^ T cells through flow cytometric analysis (Fig. 5f and Supplementary Fig. 4c). However, we did observe a notable increase in the proportions of eEF2K KO CD4^+^ T cells and eEF2K KO IL-17A^+^CD4^+^ T cells in comparison to their WT counterparts. We surmised that absence of eEF2K showed higher inflammation and more severe arthritis development.

**Fig. 6:**
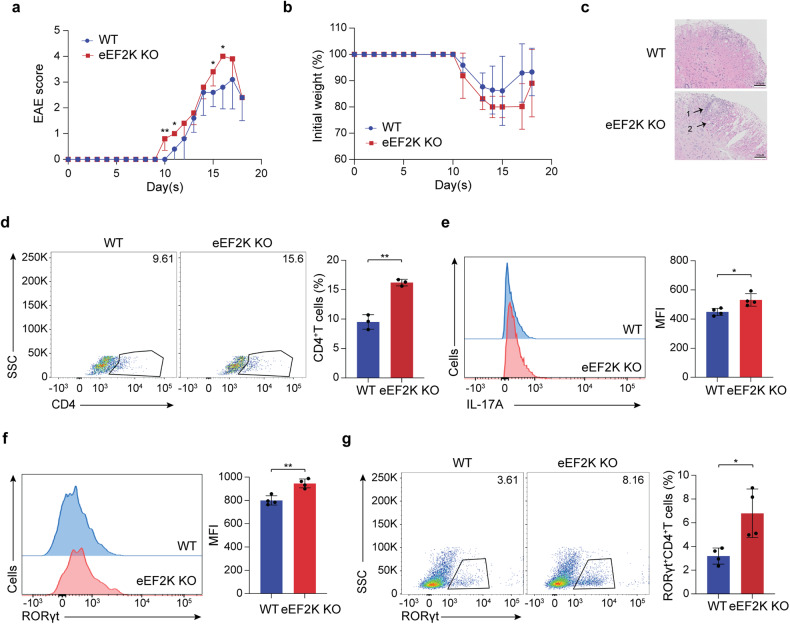
The absence of eEF2K exacerbates multiple sclerosis in the experimental autoimmune encephalomyelitis (EAE) model. Both WT and eEF2K KO mice received subcutaneous injections of an emulsion containing complete Freund’s adjuvant (CFA) and MOG_35-55_ in the upper and lower back regions. Subsequently, they were administered intraperitoneal injections of pertussis toxin, with the first injection occurring 2 h after the initial emulsion injection and the second injection administered 22–26 h later. **a** The EAE score was measured blindly daily (*n* = 5 mice in each group). **b** Mice weights were measured daily (*n* = 5 mice in each group). **c** Representative images of H&E staining of spinal cords isolated from WT and eEF2K KO mice with EAE. Arrows showed (1) inflammatory cell infiltration and (2) demyelination. Scale bar, 100 μm. **d** Representative results of the surface FACS analysis of CD4^+^ T cells in WT and eEF2K KO T cells from the brains of the mice with EAE. **e** Intracellular FACS analysis of IL-17A in WT and eEF2K KO T cells from the brains of the mice with EAE (*n* = 4). Summary MFI was presented as mean ± SD in the bar graph. **f** Intracellular FACS analysis of RORγt in WT and eEF2K KO CD4^+^ T cells from the brains of the mice with EAE (*n* = 4). Summary MFI was presented as mean ± SD in the bar graph. **g** Intracellular FACS analysis of RORγt in WT and eEF2K KO CD4^+^ T cells from the spleens of the mice with EAE (*n* = 4). Summary MFI was presented as mean ± SD in the bar graph. *P < 0.05; **P < 0.01; ***P < 0.005; ****P < 0.001, unpaired *t*-test

A heightened population of Th17 cells has been detected within the central nervous system (CNS) lesions of individuals with multiple sclerosis, a condition in which immune cells target the CNS, leading to demyelination and subsequent paralysis and walking disability.^35^ To investigate the potential influence of eEF2K on multiple sclerosis (MS), we established an experimental autoimmune encephalomyelitis (EAE) model. The EAE model is particularly well-suited for examining the autoimmune mechanisms underlying MS, making it a valuable tool for investigating experimental treatments.^7^ Both WT and eEF2K KO mice were subjected to subcutaneous immunization using myelin oligodendrocyte glycoprotein (MOG_35-55_), which was suspended in an emulsion with complete Freund’s adjuvant (CFA), along with intraperitoneal injections of pertussis toxin to induce EAE. Mice that develop EAE typically display symptoms such as a drooping or limping tail and hindlimb paralysis. The eEF2K KO group with EAE exhibited an earlier onset of the disease and displayed a more severe disease score compared to the control group (Fig. 6a). The heightened EAE score showed a negative correlation with the mice’s weight,^36^ although no statistically significant difference was observed (Fig. 6b). The increased severity of EAE in eEF2K KO mice was accompanied by a greater infiltration of inflammatory cells and more extensive demyelination in the spinal cords (Fig. 6c). Consistently, analysis of the T cells that infiltrated the brain showed a significantly increased frequency of CD4^+^ T cells (Fig. 6d), particularly those expressing RORγt and producing the IL-17A cytokine (Fig. 6e, f). Furthermore, there was a greater population of Th17 cells observed in the spleens of the eEF2K KO group with EAE in comparison to the WT group with EAE (Fig. 6g). Taken together, the data indicate that the deficiency of eEF2K promotes Th17 cell responses in inflammation-related diseases, including rheumatoid arthritis and multiple sclerosis.

**Fig. 7:**
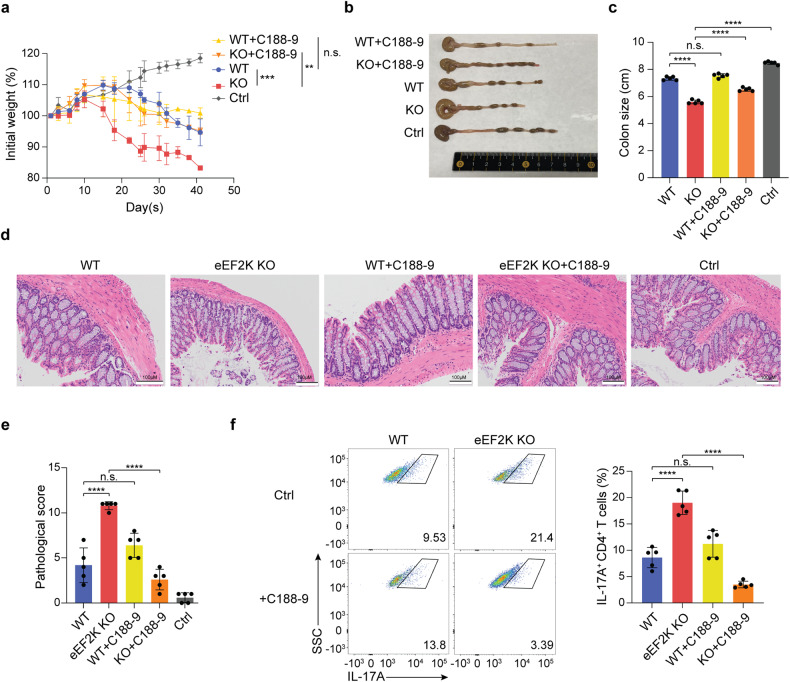
The absence of eEF2K activates STAT3 and facilitates inflammation-related diseases in the T-cell transfer colitis model. NSG mice, which received CD4^+^CD25^-^CD45RB^hi^ cells from either WT or eEF2K KO donors, were treated with the STAT3 inhibitor, C188-9. The initial weight changes in NSG mice following T cell transfer were monitored. The experiments were concluded six weeks after the initiation of T cell transfer, as mandated by the animal study protocol, due to a substantial body weight loss exceeding 20% in the mice. **a** Percentage of initial weight among groups (WT, KO, WT with C188-9, KO with C188-9, and control) was shown. Ctrl group is the NSG mice without T cells and the C188-9 treatment. (*n* = 5 mice per group). **b** Representative images of colons among groups (WT, KO, WT with C188-9, KO with C188-9, and control) were shown. **c** Results of colon length in **b** were shown with the standard deviations (*n* = 5 mice per group). **d** Representative H&E-stained colons of all groups were imaged. Scale bars are 100 μm. **e** The quantification of pathological scores was determined based on the results from **d**. **f** Lamina Propria cells were first gated with a CD4 marker and examined IL-17A^+^CD4^+^T cell populations. Representative flow cytometry plot and a bar graph showing cell frequencies of IL-17A^+^CD4^+^T cells in indicated mice (*n* = 5 mice per group). Summary data were presented as mean ± SD, derived from 3 independent experiments. *P < 0.05; **P < 0.01; ***P < 0.005; ****P < 0.001, unpaired *t*-test

To further investigate whether eEF2K-mediated alteration of CD4^+^ T cells is the main contributor to the development of inflammation-related diseases and to exclude the whole knockout mice effects, we used a T cell transfer colitis model, a suitable experimental model for studying ulcerative colitis related to CD4^+^ T cell-based pathogenesis.^37^ In these experiments, naive WT or eEF2K KO CD4^+^ T cells (CD4^+^CD25^-^CD45RB^hi^ naive T cells) were introduced intravenously into NSG mice, which lack mature T cells, B cells, and NK cells.^38^ The mice that received these inflammatory CD4^+^ T cells consistently experienced weight loss and developed colitis within a period of 4 to 8 weeks. NSG mice receiving eEF2K KO CD4^+^ T cells (eEF2K KO group) underwent significant weight losses, as compared to the WT group (Fig. 7a), and colitis was more severe in the eEF2K KO group than in controls, as evidenced by their shortened colons (Fig. 7b, c). The experiments were terminated 6 weeks after the onset of T cell transfer due to significant body weight loss (>20%) of the mice in the eEF2K KO group in accordance with the animal study protocol. Histological examination using H&E staining of the colons collected 6 weeks after T cell transfer revealed more severe inflammation in the eEF2K KO group compared to the WT group. This was evidenced by increased inflammatory cell infiltration and disrupted crypt structure (Fig. 7d, e). Flow cytometry analysis demonstrated that, after the development of colitis, the eEF2K knockout group had higher populations of IL-17A^+^ CD4^+^ T cells in the colons compared to the WT group (Fig. 7f). These experiments collectively showcased that the lack of eEF2K resulted in an inflammatory microenvironment, exacerbated inflammation-related diseases, promoted colitis, multiple sclerosis, and arthritis through dysfunctional CD4^+^ T cells, and increased the secretion of pro-inflammatory cytokines.

### The deficiency of eEF2K upregulates STAT3 and exacerbates inflammation-related diseases in the T-cell transfer colitis model

The increase in STAT3 expression in eEF2K knockout CD4^+^ T cells (Fig. 4b, c) prompted us to explore the role of this transcription factor in the eEF2K-mediated regulation of colitis. To begin, we established the T-cell transfer colitis model by intravenously (*i.v*.) injecting naive CD4^+^CD25^-^CD45RB^hi^ T cells from either WT or eEF2K KO mice into NSG mice. Subsequently, we treated these mice with the STAT3 inhibitor, C188-9, through intraperitoneal (*i.p*.) injections every 2 days for a duration of 2 weeks. All the mice receiving the naive CD4^+^ T cells developed colitis, as indicated by the substantial loss of body weight, histologically evident damage, and a reduction in colon size (Fig. 7). Remarkably, while C188-9 treatment significantly alleviated the severity of colitis in the eEF2K KO group, this STAT3 inhibitor did not affect the severity of the disease in the WT group, as determined by body weight (Fig. 7a), colon size (Fig. 7b, c), and pathologic score (Fig. 7d, e). Flow cytometric analysis showed that the IL-17A^+^CD4^+^ T cell population, collected from both colons and spleens, significantly decreased when the KO group was treated with C188-9, compared with the KO group without C188-9 treatment (Fig. 7f, g). We observed no difference in the WT group, with or without C188-9 treatment (Fig. 7f, g). Collectively, these results demonstrate that eEF2K regulation of the STAT3 pathway in controlling inflammatory responses.

## Discussion

While eEF2K has been recognized as a crucial component of CD8^+^ cytotoxic T lymphocytes, its role in modulating the function and activities of effector CD4^+^ T cells has remained undefined. To the best of our knowledge, this is the first report elucidating the role of eEF2K in regulating CD4^+^ T cell activities and its involvement in controlling inflammation-related diseases. The data presented herein demonstrate that eEF2K deficiency in T cells leads to the dysregulated production of inflammatory cytokines in CD4^+^ T cells, resulting in a Th17 profile following antigenic stimulation (Fig. 1). Additionally, we observed that the loss of this kinase exacerbates inflammation in in vivo murine models of arthritis, multiple sclerosis, and colitis.

Indeed, there have been reports indicating that silencing eEF2K expression results in reduced production of ROS in vascular smooth muscle cells and human colon cancer cells. This suggests that eEF2K can play a role in regulating ROS production in different cellular contexts.^20,23,39^ These observations indicate that higher levels of ROS production results in higher inflammatory responses and ultimately causes more severe disease. However, we found that loss of eEF2K increases ROS production, contradicting these observations. The cause for the discrepancy requires further investigation. STAT3 serves as a primary transcription factor for Th17 cells in the mitochondria as well as in the cytoplasm.^40^ Recent studies revealed that STAT3-dependent mechanisms are required for T cell metabolism and function.^41^ The relationship between STAT3 and eEF2K has indeed been a subject of some controversy, and it appears to vary depending on the cell type and context. Some recent reports suggest that eEF2K forms a complex with STAT3 in lung cancer cells, and the loss of eEF2K can result in decreased expression of STAT3. Such interactions and regulatory mechanisms can be complex and context-dependent, and further research is needed to fully understand the intricacies of their relationship in various biological settings.^42,43^ It is worth noting that the relationship between eEF2K and STAT3 can indeed vary across different cell types. In some cases, eEF2K may upregulate the levels of STAT3, as observed in various cell types, including melanoma. These complexities highlight the multifaceted nature of cellular signaling pathways and the need for careful consideration of specific conditions and cell types when studying their interactions. Researchers often need to examine these interactions in the specific cellular and disease contexts of interest to gain a more comprehensive understanding.^44^ While this study did not directly investigate the role of STAT3 in eEF2K knockout (KO) CD4^+^ T cells, it did highlight a significant increase in STAT3 expression in these cells. This observation is noteworthy as it suggests a potential link between eEF2K and STAT3 in the context of CD4^+^ T cell function. Furthermore, these findings align with emerging evidence indicating an increased population of Th17 cells in arthritic patients, suggesting a possible significant role for these cells in the development of arthritis. This suggests that the dysregulation of eEF2K and its impact on CD4^+^ T cell function, including the promotion of Th17 profiles, may contribute to the pathogenesis of diseases like rheumatoid arthritis. Further research may provide more insights into the complex interplay between eEF2K, STAT3, and CD4^+^ T cells in autoimmune disorders.^45^ Our work provides a link between the loss of eEF2K in CD4^+^ T cells leading to a shift towards the Th17 profile and the development of inflammation-related diseases, including rheumatoid arthritis.

Activated CD4^+^ T cells undergo substantial changes in the process of protein synthesis, particularly in the energetically demanding phase of translation. These adaptations are essential to meet the increased demands for cell growth, proliferation, and responding to nutrient availability during activation.^46^ Our findings suggest that the absence of eEF2K in CD4^+^ T cells leads to abnormal metabolic processes, including glycolysis and oxidative phosphorylation. This altered metabolism may result in an accumulation of excess proteins, which could potentially be converted into glucose or other energy-building blocks. These resources may then be utilized to support the production of a substantial number of cytokines and provide the necessary energy for rapid cell growth when eEF2K is lacking in CD4^+^ T cells. This highlights the intricate relationship between cellular metabolism and immune responses, particularly in the context of CD4^+^ T cell function and cytokine production. Our research indicates that the absence of eEF2K leads to an increase in ROS production, likely resulting from the elevated oxidative stress associated with eEF2K deficiency. Interestingly, we observed an augmentation in mitochondrial mass in eEF2K-depleted cells. This suggests that the substantial increase in OXPHOS and ROS production may not solely be attributed to the loss of eEF2K but could also be influenced by the higher mitochondrial mass in eEF2K knockout CD4^+^ T cells. Furthermore, while our study primarily focused on the effects of eEF2K on CD4^+^ T cells and arthritis, it’s important to consider that other immune cell types may potentially impact the severity of arthritis as well. The immune system operates as a complex network of interactions, and the contributions of various immune cell subsets can play critical roles in the development and progression of inflammatory diseases like arthritis. Future research may delve further into these interactions to provide a more comprehensive understanding of the mechanisms at play. In this study, we employed an EAE model and a T cell-transfer colitis model. We successfully replicated our in vitro findings in animal models of inflammation-related diseases, such as arthritis, multiple sclerosis, and colitis. These results underscore the pivotal role of eEF2K in preserving immunological balance and exacerbating inflammation when eEF2K was lacking.

Our data reveal a significant increase in total STAT3 expression within eEF2K-deficient CD4^+^ T cells. This upregulation of STAT3 levels subsequently triggers abnormal mitochondrial activity and impairs the functionality of CD4^+^ T cells, ultimately contributing to a microinflammatory milieu. In our forthcoming research, we intend to delve deeper into the intricate interplay between eEF2K and STAT3, which promises to enrich our narrative and provide a more profound understanding of the underlying mechanisms at play. Overall, our findings strongly indicate the functional indispensability of eEF2K in inflammation-related diseases. Boosting eEF2K levels in CD4^+^ T cells to curtail the proinflammatory microenvironment could potentially usher in a novel approach for the prevention and treatment of inflammatory conditions, such as rheumatoid arthritis, multiple sclerosis, and ulcerative colitis.

**Fig. 8:**
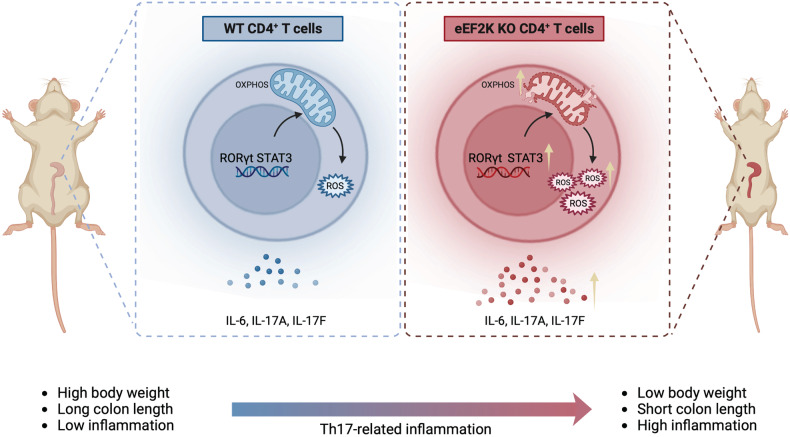
eEF2K-mediated control of CD4^+^ T cells restrain inflammatory diseases. Mice deficient in eEF2K exhibited an elevated metabolic profile and enhanced production of pro-inflammatory cytokines. eEF2K was found to modulate mitochondrial bioenergetics and redox balance by influencing STAT3, thereby exerting an impact on CD4^+^ T cell function. The depletion of eEF2K led to the development of Th17-related autoimmune diseases, such as colitis, through the STAT3 pathway. This figure was created with BioRender.com

In summary, this study has unveiled the pivotal role of eEF2K in regulating CD4^+^ T cell functions and highlights how its absence steers these cells toward a Th17 profile, with mitochondrial function and metabolism playing a central role in this process. Furthermore, our findings illustrate that the deficiency of eEF2K in CD4^+^ T cells exacerbates inflammation-related diseases by promoting the Th17 profile (Fig. 8).

## Materials and methods

### Mice

C57BL/6 congenic mice (B6 Thy1.2; Thy1.1^+^) and NSG (NOD-scid IL2Rg^null^) mice were attained from The Jackson Laboratory (Bar Harbor, Maine, USA). eEF2K^-/-^ mice (C57BL/6 background; Thy1.2^+^)^47^ were generous gifts from Dr. Alexey Ryazonov (Rutgers University, Robert Wood Johnson Medical School, New Jersey). All mice were maintained under pathogen-free conditions, with a 12:12-hour light-dark cycle, within the animal facility of the Texas A&M University. NSG (NOD-scid IL2Rg^null^). C57BL/6 congenic mice and eEF2K deficient mice (6–8 weeks, male) were used for the murine CIA model. NSG (NOD-scid IL2Rg^null^) mice were used for the T cell transfer colitis model. C57BL/6 congenic mice and eEF2K deficient mice (9–13 weeks, female) were used for the multiple sclerosis model. All mice used for the rest of the experiments were male and female, aged 5–10 weeks.

### Ethics statement

All animal studies were performed in accordance with the guidelines described by Institutional Animal Care and Use Committee (IACUC no. 2018-0065), Texas A&M University. All the mice were sacrificed by euthanasia.

### Cell culture

All cells were cultured in RPMI1640 medium (#11875093) supplemented with 10% heat-inactivated fetal bovine serum (#A3840001), 2 mM GlutaMAX (#35050061), 1 mM sodium pyruvate (#11360070), 1% MEM nonessential amino acids (#M7145-100mL), 10 mM HEPES (#15630080), 55uM 2-Mercaptoethanol (#21985023), and 1% penicillin-streptomycin (#15070063). All reagents were attained from Thermo Fisher Scientific (Waltham, MA, USA).

### CD4^+^ T cell purification, expansion, and Th1, Th2, and Th17 differentiation in vitro

Naive CD4^+^ T cells were isolated from the spleens and lymph nodes (LNs) of mice using a magnetic bead separation technique provided by the naive CD4^+^ T cell isolation kit (#480040).

Naive CD4^+^ T cells (1 × 10^6^ cells/mL) were activated by exposure to anti-CD3 monoclonal antibody (mAb; 4 µg/mL, #100340) and anti-CD28 mAb (4 µg/mL, #102116) for a duration of three days, in preparation for subsequent experiments. All the reagents used were sourced from BioLegend (San Diego, CA, USA).

To induce Th1 differentiation, naive CD4^+^ T cells were stimulated with plate-coated anti-mouse CD3 (4 µg/mL; #100339), anti-mouse CD28 (4 µg/mL; #102116), along with Th1-associated cytokines, including anti-mouse IL-4 (10 µg/mL; #504122), recombinant mouse IL-2 (5 ng/mL; 575402), and recombinant mouse IL-12 (10 ng/mL; #577002), for a period of four days. All cytokines used in this experiment were sourced from BioLegend (San Diego, CA, USA).

To induce Th2 differentiation, naive CD4^+^ T cells were activated with plate-coated anti-mouse CD3 (4 µg/mL; #100339), anti-mouse CD28 (4 µg/mL; #102116), in the presence of Th2-associated cytokines, which included recombinant mouse IL-2 (20 ng/mL; #575402) and recombinant mouse IL-4 (50 ng/mL; #574302), for a duration of four days. All cytokines used in this experiment were obtained from BioLegend (San Diego, CA, USA).

To induce Th17 differentiation, naive CD4^+^ T cells were activated with plate-coated anti-mouse CD3 (4 µg/mL; #100339) and anti-mouse CD28 (4 µg/mL; #102116), along with Th17-associated cytokines, which included anti-mouse IL-4 (10 µg/mL; #504122), anti-mouse IL-6 (50 ng/mL; #575704), anti-mouse IL-23 (5 ng/mL; #589002), and anti-human TGF-β (1 ng/mL; #781802), for a duration of four days. All cytokines utilized in this experiment were procured from BioLegend (San Diego, CA, USA).

### Antibodies (Abs) and dyes

The antibodies and reagents used in this study were as follows. For CD4^+^ T cell stimulation, Th1, Th2, Treg, and Th17 differentiation: Anti-CD3 monoclonal antibody (mAb; 4 µg/mL; #100340), Anti-CD28 monoclonal antibody (mAb; 4 µg/mL; #102116), Recombinant mouse IL-2 (5 ng/mL; #575402), Anti-mouse IL-4 (10 µg/mL; #504122), Recombinant mouse IL-4 (50 ng/mL; #574302), Anti-mouse IL-6 (50 ng/mL; #575704), Recombinant mouse IL-12 (10 ng/mL; #577002), Anti-mouse IL-23 (5 ng/mL; #589002), Anti-human TGF-β (1 ng/mL; #781802). All the above antibodies and cytokines were sourced from BioLegend (San Diego, CA, USA). For intracellular and cell-surface staining: Brilliant Violet 711 anti-mouse T-bet (#644819), APC anti-mouse GATA3 (#653805), Pacific Blue anti-mouse FOXP3 (#118903), APC anti-mouse IL-6 (#504507), PE anti-mouse IL-17A (#506903), Alexa Fluor 488 anti-mouse IL-17F (#517005), PE anti-mouse IL-17F (#517007), Alexa Fluor 700 anti-mouse IL-17A (#506914), Pacific Blue anti-mouse IL-17A (#506917), APC anti-mouse IL-17A (#506915), APC anti-mouse IL-6 (#504507), PE/Cyanine7 anti-mouse CD69 (#104512), PE STAT-3 (#678007), Brilliant Violet 785 anti-mouse IFN-γ (#505837), FITC anti-mouse CD4 (#100406), APC anti-mouse CD4 (#100411), PE anti-mouse CD8 (#155008), FITC anti-mouse CD8 (#155004), Brilliant Violet 421 anti-mouse IL-23R (#150907). Additionally, PE anti-mouse RORγt (#562607) was obtained from BD Bioscience.

For apoptosis detection: PE Annexin V apoptosis detection kit with 7-AAD (#640934) from BioLegend (San Diego, CA, USA). For immunofluorescence: eEF2K (#PA5-22175), MitoTracker Deep Red FM (#M22426), Alexa Fluor 488 goat anti-mouse IgG1 (#A-21121), Invitrogen prolong gold antifade mountant with DAPI (#P36941). All the immunofluorescence reagents were from Thermo Fisher Scientific (Waltham, MA). For Western blotting: Anti-pY705-Stat3 (#651001), Anti-STAT3 (#678001), β-actin (#664802) from BioLegend (San Diego, CA, USA), Anti-pY705-Stat3 (#9145 T) and anti-phospho-STAT3 (Ser727) (#9134) from Cell Signaling Technology (Danvers, MA). Secondary antibodies for Western blots: HRP goat anti-mouse IgG (#405306), HRP donkey anti-rabbit IgG (#406401), HRP goat anti-rat IgG (#405405). All secondary antibodies were sourced from BioLegend (San Diego, CA, USA). For measuring CD4^+^ T cell proliferation: Carboxyfluorescein succinimidyl ester (CFSE; #C34554) from Thermo Fisher Scientific (Waltham, MA, USA).

### Metabolism analysis

T cell metabolism, encompassing lactate production, glycolysis, and oxidative phosphorylation, was assessed using the Lactate-Glo Assay kit (#J5021) and Glucose Uptake-Glo Assay kit (#J1341) obtained from Promega (Madison, WI, USA). The Seahorse Extracellular Flux Analyzer XFp kits and instrument were procured from Agilent Technologies (Santa Clara, CA, USA). For the Lactate-Glo Assay and Glucose Uptake-Glo Assay, CD4^+^ T cells were cultured in vitro for one day and then stimulated with anti-CD3 and anti-CD28 monoclonal antibodies, following the manufacturer’s instructions. For real-time extracellular flux assays, CD4^+^ T cells were cultured in vitro for three days in RPMI medium. A day before the assay, the cell culture medium was replaced with Seahorse XF RPMI Base Medium (#103336-100). Activated CD4^+^ T cells (1 × 10^5^ per well) were plated in Cell-TakTM-coated Seahorse 96-well plates and pre-incubated at 37 °C for 60 min without carbon dioxide. The growth medium was then substituted with Seahorse XF DMEM with pH 7.4, which contained 1 mM pyruvate, 2 mM glutamine, and 10 mM glucose. For the glycolysis assessment (XFp glycolysis stress test kit, #103017-100), 0.5 µM rotenone/antimycin A and 50 µM 2DG were added to the cell culture, and the extracellular acidification rate (ECAR) was measured. To evaluate oxidative phosphorylation (XF Cell Mito Stress Test Kit, #103010-100), 1 μM oligomycin, 2 μM FCCP, and 0.5 μM rotenone/antimycin A were introduced to the cell culture, and the oxygen consumption rate (OCR) was measured.

### ROS detection assay

To assess intracellular reactive oxygen species (ROS), we conducted the DCFDA/H2DCFDA assay (#ab113851) obtained from Abcam (Waltham, MA, USA). In this assay, both WT and eEF2K KO CD4^+^ T cells were activated by anti-CD3 monoclonal antibody and anti-CD28 monoclonal antibody for a duration of three days. On the third day, the cells were stained with DCFDA/H2DCFDA and incubated at 37 °C for 30 min. To investigate mitochondrial ROS, we utilized the MitoSOX Red mitochondrial superoxide indicator (#M36008) supplied by Thermo Fisher Scientific (Waltham, MA, USA). Mitochondrial ROS staining was performed on day 3 after activation, involving a 37 °C incubation for 30 min.

### FACS analysis

The cells were resuspended in a cell staining buffer and then incubated with surface-marker antibodies for 30 min at 4°C in the dark. For intracellular staining, cells were restimulated with a 1X cell activation cocktail (#423301) 4 h before intracellular staining. In the case of cytokine detection, such as IL-2, IFN-γ, and IL-17A, a protein transport inhibitor, brefeldin A (#420601), was added 4 h prior to intracellular staining. To ensure accurate results, cells were first stained with the Zombie NIR fixable viability kit (#423105), followed by fixation with fixation buffer (#420801), and subsequent permeabilization using intracellular staining perm wash buffer (#421002), all following the manufacturer’s guidelines. All these reagents were procured from BioLegend (San Diego, CA, USA). The fluorophore-conjugated antibodies were introduced into the cell suspensions and incubated for 20 min in the dark at room temperature. Flow cytometry analysis was carried out using a BD-FACS Fortessa instrument (BD Biosciences, San Diego, CA, USA), and the data obtained were subsequently analyzed with FlowJo software (TreeStar, OR, USA).

### In vitro inhibition of ROS or STAT3

To counteract the effects of ROS, we introduced the ROS inhibitor NAC (5 mM, #A7250-10G) into the T cell media along with the cells on day 0. To inhibit STAT3 activity, cells were pre-treated with the STAT3 inhibitor C188-9 (#573128) for 1 h at 37 °C. Both inhibitors were procured from Sigma-Aldrich (St. Louis, MO, USA). As a control, an equivalent concentration of DMSO was administered, matching the concentration of the highest dose of compound used in the assay (0.15% to 0.5%). For in vivo experiments, we administered 0.275 mg of C188-9 (dissolved in 15 µL of DMSO) intraperitoneally to mice developing colitis every 2 days. The control group of mice received an equivalent volume of DMSO.

### Immunofluorescent microscopy

CD4^+^ T cells were cultured on coverslips, which were positioned within a 12-well plate. To facilitate cell adhesion to the coverslips, centrifugation was employed, after which the supernatant was carefully removed. Subsequently, the cells were fixed with ice-cold methanol. Following fixation, the cells were incubated with primary antibodies, succeeded by secondary antibodies. Finally, to preserve the specimens, the cells on the coverslips were mounted using Prolong Gold antifade mountant (#P36930) from Thermo Fisher Scientific (Waltham, MA, USA). The cells on the coverslips were examined using an Olympus FV3000 Confocal Microscope.

### Western blotting

The cells were collected and lysed using M-PER Mammalian Protein Extraction Reagent (#78503). To inhibit protease activity, a protease inhibitor cocktail (#P178437) was added to the lysis buffer. Protein lysates were quantified utilizing the bicinchoninic acid (BCA) protein assay kit (#23225), following the manufacturer’s instructions. Whole-cell lysates were separated by running them through an SDS-PAGE gel. Subsequently, the samples were transferred onto polyvinylidene difluoride (PVDF) membranes. The primary antibody was incubated on the PVDF membrane overnight at 4°C, followed by incubation with the secondary antibody for 1 h at room temperature. The reactions were detected using Pierce ECL Western Blotting Substrate (#32106). All the reagents used were sourced from Thermo Fisher Scientific (Waltham, MA, USA).

### Global proteomics analysis

Cell lysates were prepared using M-PER buffer, as previously described. Subsequently, the cell lysates were separated via SDS-PAGE gel electrophoresis. Once the electrophoresis was completed, the gel was sectioned into pieces. These gel fragments were subjected to washing with acetonitrile (CAN) and then treated with DTT to facilitate reduction. In-gel digestion was carried out using a trypsin solution, and the resulting extracts were collected for subsequent analysis. The analysis was performed using LC-MS/MS on an Orbitrap Fusion Tribrid mass spectrometer, provided by Thermo Fisher Scientific (Waltham, MA, USA).

### Quantitative Real-Time PCR

Total RNA extraction was accomplished using the RNeasy Mini Kits (#74104) by QIAGEN (Germantown, MD, USA). Subsequently, complementary DNA (cDNA) synthesis was carried out using a high-capacity cDNA reverse transcription kit (#4368814) from Thermo Fisher Scientific (Waltham, MA, USA).

The primers employed for detecting GAPDH and mitochondrial copy numbers were adapted from previous studies^48,49^ with some modifications. Additionally, the primers utilized for detecting RORγt and β-actin were derived from literature sources.^50–52^ The specific primer sequences were as follows:

Mitochondrial copy number forward primer: 5’-CTAGAAACCCCGAAACCAAA-3’

Mitochondrial copy number reverse primer: 5’-CCAGCTATCACCAAGCTCGT-3’

RORγt forward primer: 5’-CCGCTGAGAGGGCTTCAC-3’

RORγt reverse primer: 5’-TGCAGGAGTAGGCCACATTACA-3’

GADPH forward primer: 5’-GTTGTCTCCTGCGACTTCA-3’

GADPH reverse primer: 5’-GGTGGTCCAGGGTTTCTTA-3’

β-actin forward primer: 5’-GACGGCCAGGTCATCACTATTG-3’

β-actin reverse primer: 5’-AGGAAGGCTGGAAAAGACC-3’

qRT-PCR was performed using the CFX96 Touch Real-Time PCR Detection System from Bio-Rad (Hercules, CA, USA).

### Induction of murine CIA model

In our murine collagen-induced arthritis (CIA) model, we employed C57BL/6 congenic mice and eEF2K deficient mice, all of which were male and aged 6–8 weeks. The model was induced by emulsifying bovine type II collagen (#20012) with an equal volume of complete Freund’s adjuvant (CFA) (#7023), both sourced from Chondrex (Redmond, WA, USA). Mice were intradermally injected with 100 µL of this emulsion, with the first immunization on day 0 and a booster injection on day 15 at the same site. To assess the progression of arthritis, we measured arthritis scores and swollen joint size every 2–3 days. These assessments were conducted by two observers who were blinded to the experimental groups. Our arthritis scoring system was adapted and modified from our previous study and the work of Teixeira et al.^53,54^ We observed four different joint types, including interphalangeal joints, metacarpophalangeal joints, and carpal and tarsal joints. All paws, including front and hind paws, were examined individually. Each paw was assigned a score on a 4-point scale: Score 0 represented normal joints, Score 1 indicated mild redness and swelling of the joints, Score 2 represented moderate redness and swelling, Score 3 indicated severe redness and swelling, and Score 4 denoted maximal inflammation affecting the entire paw. Mice were humanely euthanized when their average clinical score reached 3. The spleens from each group of mice were dissected for flow cytometry analysis to measure various T cell populations.

### Induction of EAE mice model

EAE model induction was executed in accordance with the manufacturer’s instructions (Hooke Kit MOG_35-55_/CFA Emulsion PTX #EK-2110). Briefly, 9–13 weeks old female C57BL/6 mice or eEF2K deficient mice were immunized with antigen (MOG_35-55_) in emulsion with complete Freund’s adjuvant (CFA) subcutaneously on the upper and lower back of the mice, followed by the injection of pertussis toxin in PBS intraperitoneally on the first day of immunization and the following day. Subsequently, the mice were observed daily to monitor the disease development. Mice foods were substituted with moist chow or gel diet for the paralysis mice. EAE clinical scores were conducted using the following criteria: score 0: no disease; score 1: limp tail; score 2: limp tail and hindlimb weakness; score 3: hindlimb paralysis; score 4: hindlimb paralysis and forelimb weakness; score 5: complete paralysis or death.

### Isolation of cells from the brain and spinal cord

The method for isolating cells from the brain and spinal cord was adapted from a previous study.^30^

Initially, the tissues were dissected into small fragments to facilitate the extraction of single cells. These tissue fragments were then treated with collagenase IV (2.5 mg/mL) for 30 min at 37 °C. Subsequently, the tissues were mechanically disrupted and filtered through 40 μm and 70 μm cell strainers. The resulting cell pellets were resuspended in HBSS buffer for subsequent staining and analysis via flow cytometry.

### Induction of T cell-transfer colitis

To create the T cell-transfer colitis model, we performed intravenous (*i.v*.) injections of 0.5 million naive CD4^+^ T cells, CD4^+^CD8^-^CD25^-^CD45RB^hi^ T cells, which were sorted from the spleens and peripheral LNs of either C57BL/6 mice or eEF2K deficient mice. These T cells were injected into NSG (NOD-scid IL2Rgnull) mice. For female donor mice, female NSG mice were used as recipients to prevent graft rejection. However, for male donor mice, there were no gender restrictions for the recipient mice (male or female).^55^ Throughout the experiment, we monitored the body weight of the mice, and we calculated the percentage weight change every two days. Mice that reached below 80% of their initial body weight were humanely sacrificed in accordance with the established protocol.

### Lamina propria cell isolation

Mouse colons were dissected and cut into 1 cm pieces within a petri dish. These colon segments were then submerged in an HBSS solution. To isolate lamina propria cells, we utilized the Lamina Propria Dissociation Kit (#130-097-410) from Miltenyi Biotec, following the manufacturer’s instructions.

### Histology sample preparation

Knee joints, colon tissues, or spinal cords from each group of mice were initially fixed using 10% formalin obtained from VWR (West Chester, PA, USA) overnight, after which they were transferred to 70% ethanol for preservation. Knee joint sections underwent decalcification using formical (#NC1146066) sourced from Fisher Scientific (Waltham, MA). The tissues extracted from knee joints were subsequently subjected to H&E staining and Safranin O staining. In contrast, samples from colon tissues and spinal cords were stained using H&E, following established protocols as previously described.^56^

### Histological evaluation

Histological slides were examined using an Olympus VS120 Slide Scanner. Knee joint sections, which were subjected to Safranin O staining, were scored by two independent investigators, both blinded to the samples, using a semiquantitative scoring system. This system ranged from 0 (indicating no depletion of proteoglycan) to 4 (representing maximal proteoglycan depletion).

For H&E staining of murine colitis, two blinded investigators assigned scores based on predefined criteria, as described previously.^57^ Six different factors were considered in determining the pathological scores, with a maximum total score of 15: colon inflammation (normal to severe, 0–3), abnormal crypt (normal to severe crypt loss, 0–3), crypt abscess (absent and present, 0–1), goblet cell loss (normal to severe, 0–3), mucosal erosion and ulceration (normal to severe, 0–3), and submucosal change (normal, 0; submucosa, 1; transmural, 2).

### Statistical analysis

To analyze the data from all groups, we conducted either paired or unpaired Student’s *t*-tests, depending on the specific experimental design. We performed these statistical analyses using GraphPad Prism V.9 (San Diego, CA, USA). A *p*-value of less than 0.05 was considered statistically significant.

### Supplementary information

Revised Supplementary Materials_Clean version

## Data Availability

All data supporting the findings of this study are available in the main text and its supplementary information. Further information is available from the corresponding authors on reasonable request.
