# Supplementary material for: Control of CD4+ T cells to restrain inflammatory diseases via eukaryotic elongation factor 2 kinase
Source: Signal Transduct Target Ther. 2023 Oct 24;8:415. doi: 10.1038/s41392-023-01648-5 (PMC10598003; doi:10.1038/s41392-023-01648-5)
Supplement: Supplementary file 1 — Revised Supplementary Materials_Clean version [file 41392_2023_1648_MOESM1_ESM.docx]

Supplementary Materials for

**Control of CD4^+^ T cells to restrain inflammatory diseases via eukaryotic elongation**

**factor-2 kinase**

#

#

Hao-Yun Peng^1, 2^, Liqing Wang^1,2^, Jugal Kishore Das^1^, Anil Kumar^1^, Darby J Ballard ^1^, Yijie Ren^1^, Xiaofang Xiong^1^, Paul de Figueiredo^1, 3^, Jin-Ming Yang^4, #^, Jianxun Song^1, #^

Correspondence to: [jus35@tamu.edu](mailto:jus35@tamu.edu) or jyang@uky.edu

**This PDF file includes:**

Figures. S1 to S4

Figure. S1.


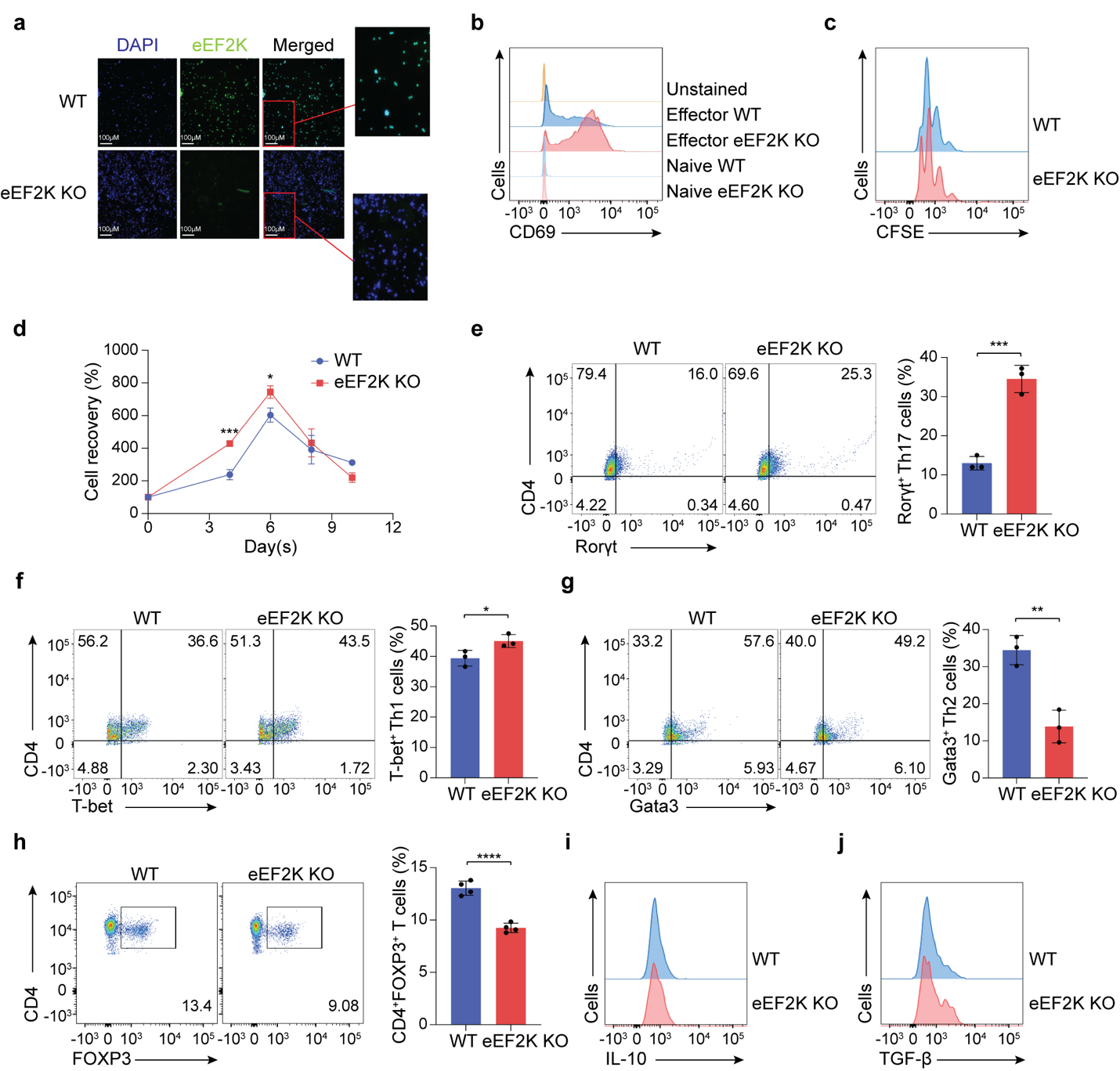


**S1. Loss of eEF2K results in abnormal effector CD4**^+^ **T cell activities and impaired inflammatory cytokines.** CD4^+^ T cells from WT and eEF2K-knockout (KO) C57BL/6 mice following 3 days *in vitro* stimulation with plate-coated with anti-CD3 plus soluble anti-CD28 antibodies (Abs). **a** eEF2K was confirmed knockout from immunofluorescent staining. Nuclei were stained with DAPI. eEF2K was stained with eEF2K antibody. Scale bar=100μm. **b** Surface FACS analysis of CD69 in the WT and eEF2K KO effector CD4^+^ T cells on day 3. WT and eEF2K KO naïve CD4^+^ T cells on day 0 were also analyzed. **c** WT and eEF2K KO CD4^+^ T cells were stained with CFSE dilution to examine their proliferative capacity. **d** Survival Curve of WT and eEF2K KO CD4^+^ T cells. **e** Effector CD4^+^ T cells were stimulated with Th17-associated cytokines, including IL-4, IL-6, IL-23, and TGFβ for 4 days. Intracellular FACS analysis of RORγt in the WT and eEF2K KO Th17 cells. Cell percentage was indicated in each quadrant, and the results of FACS analysis in the flow chart were shown in the bar graph, with the standard errors (n=3: technical replicates). **f** CD4^+^ T cells were cultured with Th1-associated cytokines for 4 days. Intracellular FACS analysis of T-bet in the WT and eEF2K KO Th1 cells was examined. Cell percentage was indicated in each quadrant, and summary data were presented as mean ± SEM, shown in the bar graph (n=3: technical replicates). **g** CD4^+^ T cells were isolated and cultured with Th2-associated cytokines for 4 days. Intracellular FACS analysis of Gata3 in the WT and eEF2K KO Th2 cells was examined. Cell percentage was indicated in each quadrant, and summary data were presented as mean ± SEM, shown in the bar graph (n=3: technical replicates). **h** Cells were obtained from WT and eEF2K KO mice. Surface and intracellular FACS analysis of CD4 and FOXP3 were examined. Treg cell percentage was indicated in each quadrant, and summary data were presented as mean ± SD, shown in the bar graph (n=4: biological replicates). **i,j** Cells were obtained from WT and eEF2K KO mice. The result of intracellular FACS analysis of IL-10 (**i**) and TGF-β (**j**) was examined after CD4^+^ and FOXP3^+^ gating. (n=3: biological replicates). *P<0.05; **P<0.01; ***P<0.005; ****P<0.001, unpaired *t*-test.

Figure. S2.


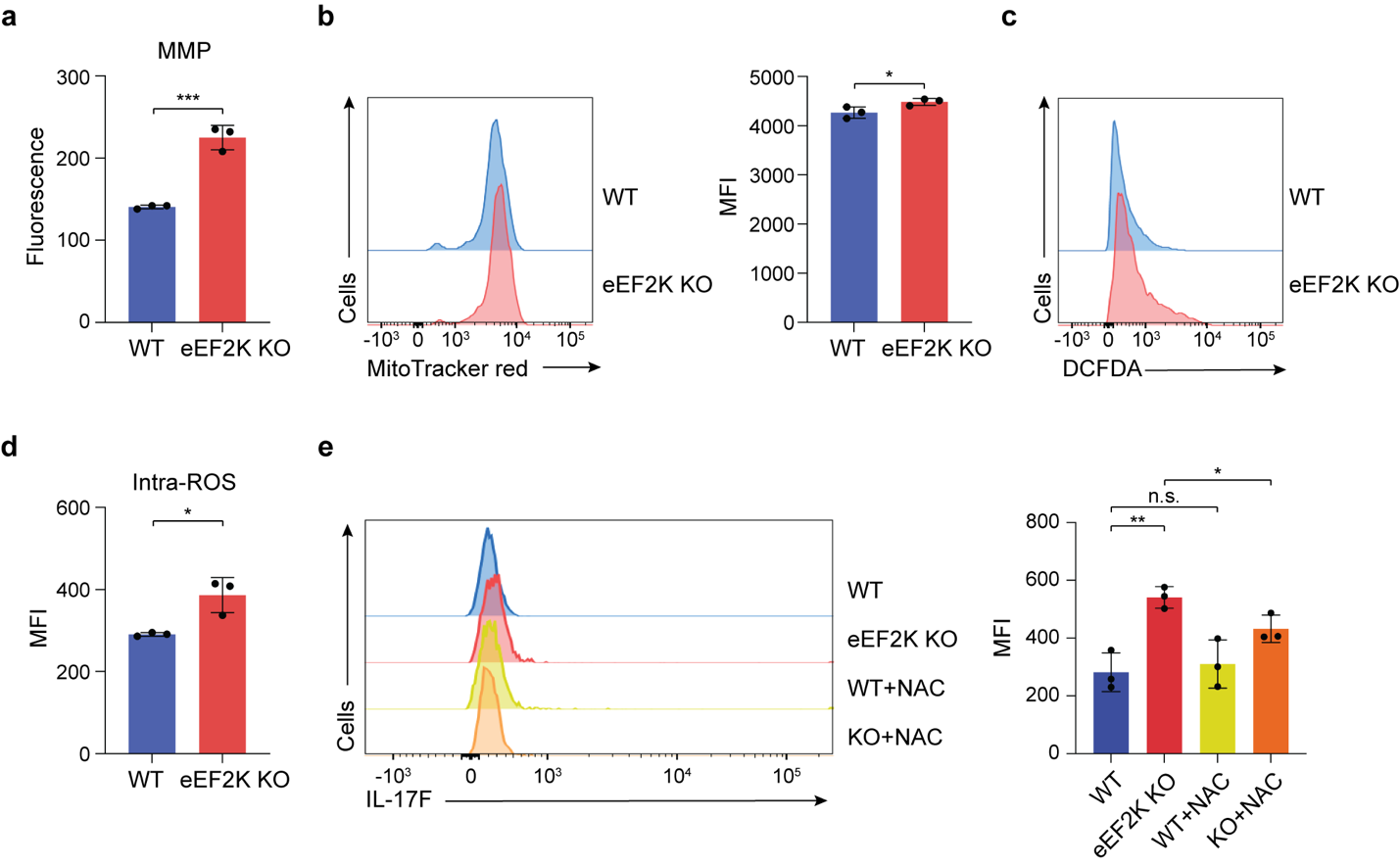


**S2. The absence of eEF2K leads to an elevated metabolic profile in Th17 cells and increased cytokine secretion in CD4**^+^ **T cells. a** WT and eEF2K KO CD4^+^ T cells were stimulated with Th17-associated cytokines and grown for 4 days. MMP was measured with JC-1 dye, as shown in JC-1 red fluorescence (n=3; technical replicates). **b** Th17-polarized cells were stained with mitoTracker Red to examine mitochondrial mass. Summarized MFI was shown in the bar graph (n=3: technical replicates). **c,d** Th17-polarized cells were stained with intracellular ROS dye-DCFDA. Summarized MFI **(d)** was shown in the bar graph (n=3; technical replicates). **e** CD4^+^ T cells were grown and were stimulated with CD3 and CD28 antibodies, with or without NAC treatment, and cultured for 3 days. Intracellular staining analysis of IL-17F was performed, and the summarized MFI was presented in the bar graph. Summary data were presented as mean ± SD, unpaired test, derived from 3 independent experiments. *P<0.05; **P<0.01; ***P<0.005; ****P<0.001, unpaired *t-*test.

Figure. S3.


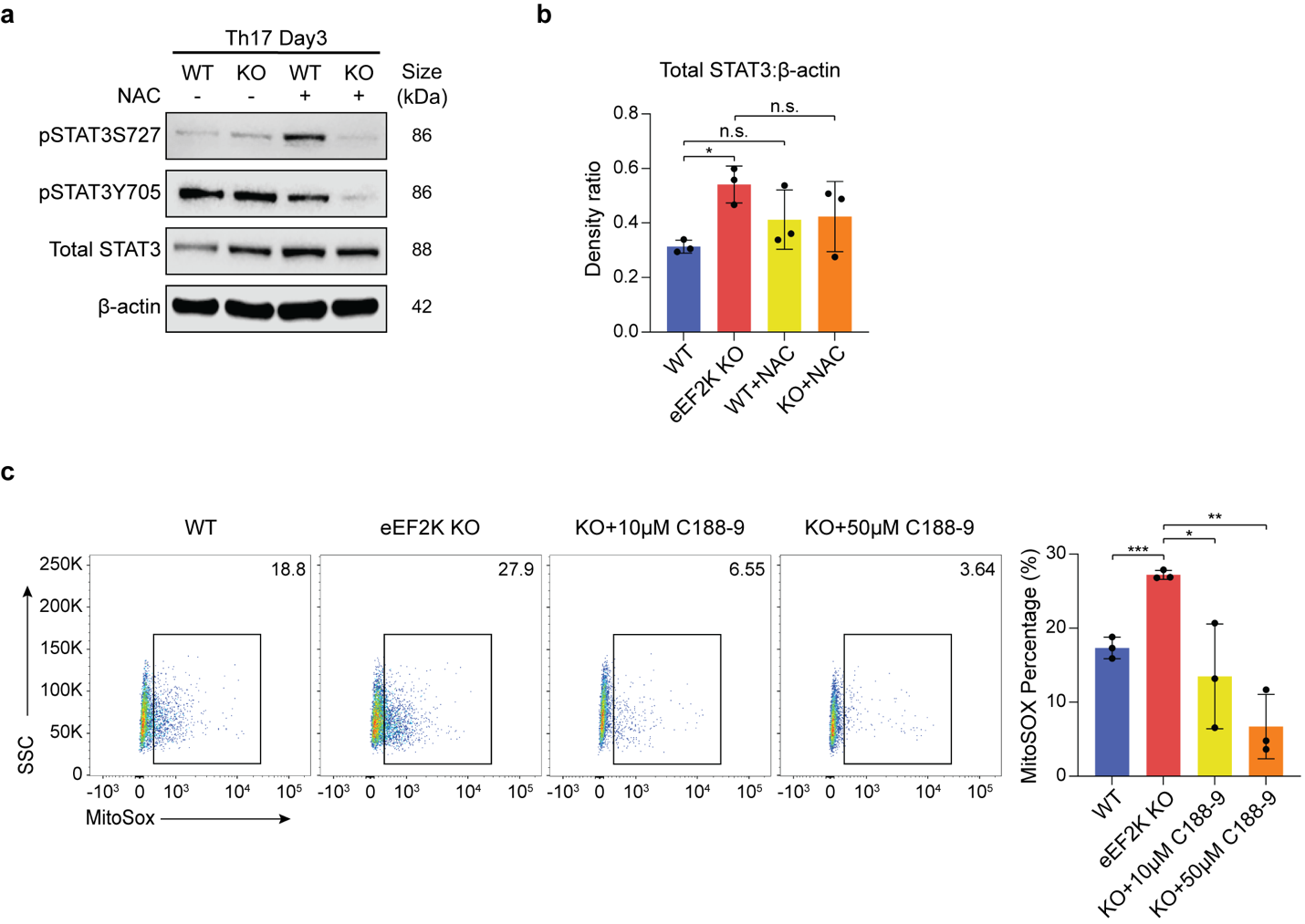


**S3. Loss of eEF2K in CD4**^+^ **T cells under Th17 culture conditions leads to up-regulation of total STAT3, and eEF2K mediates ROS production through STAT3. a** Results of the immunoblot analysis of the phospho (p)-STAT3 S727, p-STAT Y705, and total STAT3 expression of WT and eEF2K KO Th17 cells. WT or eEF2K KO naïve CD4^+^ T cells were stimulated with Th17-associated cytokines for 4 days and treated with or without NAC. All samples were subjected to immunoblotting. **b** Density ratios of total STAT3**/**β**-**actin of WT and eEF2K KO Th17 cells were assayed. Summary data were presented as mean ± SD, derived from 3 independent experiments, and analyzed with paired *t*-test. **c** Effector WT and eEF2K KO CD4^+^ T cells were treated or non-treated with different concentrations of C188-9 and examined mitochondrial ROS production was by staining with mitoSOX and analyzing through FACS. Summary data were presented as mean ± SD, unpaired *t*-test, derived from 3 independent experiments. *P<0.05; **P<0.01; ***P<0.005.

**Figure. S4.**


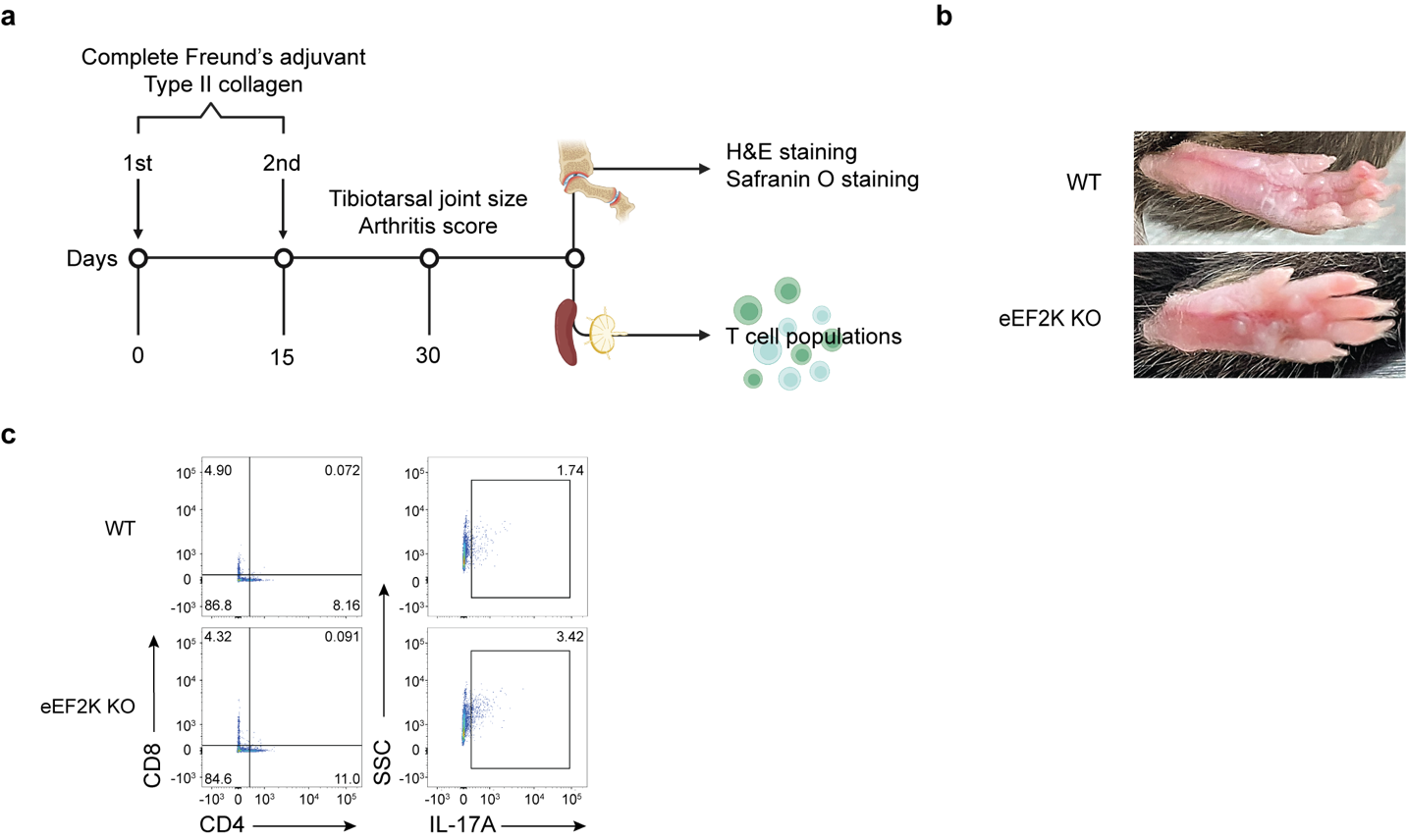


**S4.** **The absence of eEF2K exacerbates inflammation-related diseases in the collagen-induced arthritis (CIA) model.** **a** Schematic figure of the CIA mice model. WT and eEF2K KO mice were injected with the emulsion of complete Freund’s adjuvant (CFA) and bovine type II collagen on day 0 and day 15. Tibiotarsal joint size and arthritis scores were measured by two investigators blindly every 2-3 days, and once the arthritis score was reached, the mice with CIA were dissected. The knee joints were sent for H&E and Safranin O staining to examine the inflammation and joint destruction. T-cell populations were obtained from spleens and lymph nodes. This figure was created with BioRender,com. **b** Representative images of tibiotarsal joints from WT and eEF2K KO group were exhibited. **c** Representative dot plots of the surface and intracellular FACS analysis of CD4, CD8, IL-17A in WT and eEF2K KO T cells.
